# *PrtT*-Regulated Proteins Secreted by *Aspergillus fumigatus* Activate MAPK Signaling in Exposed A549 Lung Cells Leading to Necrotic Cell Death

**DOI:** 10.1371/journal.pone.0017509

**Published:** 2011-03-11

**Authors:** Haim Sharon, David Amar, Emma Levdansky, Gabriel Mircus, Yana Shadkchan, Ron Shamir, Nir Osherov

**Affiliations:** 1 Department of Clinical Microbiology and Immunology, Sackler School of Medicine, Tel-Aviv University, Ramat-Aviv, Tel-Aviv, Israel; 2 Department of Computer Science, Tel-Aviv University, Ramat-Aviv, Tel-Aviv, Israel; University of Aberdeen, United Kingdom

## Abstract

*Aspergillus fumigatus* is the most commonly encountered mold pathogen of humans, predominantly infecting the respiratory system. Colonization and penetration of the lung alveolar epithelium is a key but poorly understood step in the infection process. This study focused on identifying the transcriptional and cell-signaling responses activated in A549 alveolar carcinoma cells incubated in the presence of *A. fumigatus* wild-type and Δ*PrtT* protease-deficient germinating conidia and culture filtrates (CF). Microarray analysis of exposed A549 cells identified distinct classes of genes whose expression is altered in the presence of germinating conidia and CF and suggested the involvement of both NFkB and MAPK signaling pathways in mediating the cellular response. Phosphoprotein analysis of A549 cells confirmed that JNK and ERK1/2 are phosphorylated in response to CF from wild-type *A. fumigatus* and not phosphorylated in response to CF from the Δ*PrtT* protease-deficient strain. Inhibition of JNK or ERK1/2 kinase activity substantially decreased CF-induced cell damage, including cell peeling, actin-cytoskeleton damage, and reduction in metabolic activity and necrotic death. These results suggest that inhibition of MAPK-mediated host responses to treatment with *A. fumigatus* CF decreases cellular damage, a finding with possible clinical implications.

## Introduction

Fungi belonging to the genus *Aspergillus* are important opportunistic pathogens of immunocompromised patients. *Aspergillus fumigatus* is the main causative agent of aspergillosis. Invasive pulmonary aspergillosis (IPA) is the most severe form of the disease, in which inhaled *Aspergillus* spores invade and colonize the lungs, and subsequently spread to other organs through the bloodstream [1]. Mortality rates in patients with IPA, including those receiving intensive antifungal treatment, remain unacceptably high (50 to 70%) [2], [3].

*A. fumigatus* is responsible for approximately 90% of human *Aspergillus* infection. It reproduces asexually by producing prodigious numbers of spores, which are disseminated by air currents. Environmental studies indicate that all humans inhale at least several hundred *A. fumigatus* conidia per day [4]. Due to their small size (2–3.5 µm), *A. fumigatus* conidia often surmount the barrier posed by the ciliary action of the airway epithelium and directly enter the alveolar sacs. In healthy alveoli, the epithelial surface consists mainly of type I and type II cells (pneumocytes). Type I cells are extremely thin and flat and cover about 95% of the alveolar surface. Type II cells are cuboid and rich in secretory granules. Below the alveolar epithelium are the basal lamina and the underlying capillary endothelium. In neutropenic patients, this delicate structure is a convenient and rapidly penetrable portal for infection.

Following inhalation and binding to the alveolar epithelium, the conidia germinate. Most of the adherent conidia are normally eliminated by innate immune mechanisms, notably by resident alveolar macrophages that phagocytose and kill the conidia and recruited neutrophils that destroy both conidia and hyphae by secretion of toxic reactive oxygen intermediates and the formation of neutrophil extracellular traps (NETs) [5],[6],[7]. In immunocompromised patients lacking these defenses, conidial germination and hyphal growth occur, frequently leading to dissemination of aspergillosis [1], [8].

Major advances have been recently made in deciphering the interaction of *A. fumigatus* with macrophages and neutrophils. These cells identify *A. fumigatus* surface antigens through binding to Toll-like receptors (TLR2 and TLR4) [9], [10], [11] and dectin 1 receptors [12], [13], [14], [15]. This recognition initiates a complex signaling cascade, including activation of MAPK signaling, the NFkB pathway, and the subsequent release of proinflammatory cytokines such as IL6, TNF-α and IL1β [9], [10], [16].

In contrast, progress in identifying the molecular mechanisms of epithelial lung cell infection has been slow. Cell-culture models and *in-vitro* binding assays have demonstrated that *A. fumigatus* conidia bind to A549 type-II-like lung epithelial cells and to proteins present in the lung basal lamina [17], [18], [19]. Following binding to the cells, about 3 to 6% of infecting conidia are internalized into actin-coated vacuoles through actin- and tubulin-dependent mechanisms [20]. Externally bound conidia germinate and begin hyphal growth, causing cytokine release, cell rounding, detachment and necrosis in the infected cells [21], [22], [23]. Addition of *A. fumigatus* culture filtrate (CF) to the cells results in a similar cellular response, suggesting that factors secreted by the fungus are involved. Borger *et al*. [21] showed that CF secreted by *A. fumigatus* induces the production of proinflammatory cytokines (IL6 and IL8) and activation of NFkB in A549 cells. Interestingly, these responses are blocked by the addition of serine-protease inhibitors to the CF, indicating serine-protease dependency. Subsequently, Kauffman *et al*. [24] demonstrated that proteases present in fungal extracts from both *A. fumigatus* and other non-aspergillus fungi interact with A549 cells, leading to morphological changes, cell desquamation, and induction of proinflammatory cytokines. We have demonstrated that secreted *A. fumigatus* proteases are responsible for depolymerization of the actin cytoskeleton of A549 cells, destruction of focal adhesions and subsequent loss of adhesion, leading to cell rounding and death [25]. Recently, we and others have generated a protease-deficient strain of *A. fumigatus* in which the transcription factor *PrtT*, a positive regulator of secreted proteases, was deleted [26], [27]. CF derived from this mutant strain shows low to undetectable levels of protease activity.

In this work, we further characterized the effects of secreted *A. fumigatus* proteases on exposed A549 cells. We elucidated the genome-wide transcriptional response and the changes in protein kinase phosphorylation patterns of lung cells in response to wild-type (WT) and Δ*PrtT* protease-deficient germinating conidia and CFs, and identified key cellular signaling proteins affecting their response.

## Materials and Methods

### Microarray experiments

A549 cells were grown at a density of 1×10^6^ cells/100 mm dish, and cultured in serum-free minimal essential medium (MEM) for 16 to 18 h before treatment. Cells were incubated for 8 h with WT *A. fumigatus* conidia (AF293), Δ*PrtT* protease-deficient conidia (AF293 background), *A. fumigatus* CF, and Δ*PrtT* protease-deficient CF. Total RNA was prepared from treated and control uninfected A549 cells with RNeasy columns (Qiagen, Hilden, Germany) as recommended by Affymetrix (Santa Clara, CA). Total RNA was reverse-transcribed (Agilent Technologies, Santa Clara, CA) and the resulting cDNA was used as a template for *in-vitro* transcription with biotin-labeled oligonucleotides. Following fragmentation, it was hybridized to the array as directed by the manufacturer (Affymetrix). We used two different arrays, the GeneChip Human Genome U133A 2.0 array and later, the Human Gene 1.0 ST array offering whole-transcript coverage. Each of the 28,869 genes is represented on the array by approximately 26 probes spread across the full length of the gene. Three independent biological experiments were performed. All data is MIAME compliant and the raw data has been deposited at the NCBI GEO website, a MIAME compliant database, as detailed on the FGED Society website (GEO accession numbers GSE 24983-5).

### Microarray computational analysis

The expression profiles were analyzed using EXPANDER, a general microarray analysis software [28]. EXPANDER supports all analytical steps, including normalization and filtering, gene clustering and differential expression analysis, and various statistical tests for gene group analysis including functional enrichment and transcription factor binding site enrichment.

In preprocessing of the microarray data, following hierarchical clustering of the samples, three outliers were removed (see Text S1), and filtering of low-intensity probes left 14,831 and 14,653 genes in the CF and conidia experiments, respectively.

Differential genes were identified between each two sets of conditions, and for each set GO functional enrichment was evaluated using TANGO (FDR/False Discovery Rate <0.1) and promoter signals were evaluated using PRIMA (*p*-value <10^−4^, uncorrected). Finally, enrichment for KEGG pathways was performed using a hypergeometric test (*p*-value <10^−4^, uncorrected). See supplemental material for further details.

### Semi-quantitative PCR validation of key genes identified in the microarray analysis

RNA was isolated from 10^6^ A549 cells with TRIzol (Sigma Chemical Co., St. Louis, MO) and treated with DNase (Ambion, Austin, TX) following the manufacturer's protocols. RNA was used to synthesize cDNA using *AffinityScript* reverse transcriptase according to the manufacturer's protocols. For RT-PCR, A549 cells were cultured on 10-cm tissue-culture plates for 24 h in Dulbecco's modified Eagle's medium (DMEM); 1 ml of TRI Reagent (Molecular Research Center, Cincinnati, OH) was added to each plate, and RNA was isolated from approx 2 million cells according to the protocol supplied by the manufacturer. Total RNA (1 µg in a total volume of 12 µl) was denatured at 70°C for 10 min and used for reverse transcription as described by the standard protocol (Stratagene). PCR amplification was performed with downstream primers specific for the human IL8 and MCP1 genes and the housekeeping gene β-actin. PCR products were analyzed by gel electrophoresis. Analysis was performed on samples collected during the logarithmic phase of the PCR. Each PCR was independently performed three times on the same RNA sample. Control DNase-treated RNA samples that lacked reverse transcriptase were PCR-amplified in parallel and showed no amplified fragments. Densitometric analysis was carried out with the TINA 2.0 software package (Molecular Dynamics Inc., Sunnyvale, CA). Densitometric values were normalized by calculating the ratio between the experimental point and actin expression. Final values were derived by calculating the ratio between the normalized experimental and reference points. Statistical analysis was performed using Student's two-tailed *t* test.

### Preparation of fungal CF

Conidia were collected in a 0.01% (vol/vol) Tween 80 (Sigma) solution, washed twice in phosphate-buffered saline (PBS), and resuspended at a concentration of 1×10^5^ conidia/ml in 100 ml of MEM containing 10% (wt/vol) fetal calf serum (FCS; Biological Industries, Beit-Haemek, Israel). The FCS was heated for 30 min at 66°C to inactivate endogenous proteases. Fungal cultures were grown in an orbital incubator for 48 h, 37°C at 200 rpm, conditions which have previously been shown to produce a toxic CF [26]. CF contains the entire range of molecules secreted by the fungus, including low molecular-weight secondary metabolites and secreted proteins. To prepare WT-CF devoid of proteolytic activity, the CF was heated at 70°C for 30 min. This treatment reduced the proteolytic activity of the CF (as measured by the azocasein assay) to undetectable levels.

### Western blot analysis for endogenous JNK, ERK1/2, p38 and *c-jun* phosphorylation

A549 cells were treated for various times with *A. fumigatus* CF, or 1×10^7^ *A. fumigatus* conidia (AF293 and Δ*PrtT* strains). Subsequently, cells were trypsinized and centrifuged at 500 g for 5 min. Cells were washed in PBS and incubated with 60 µl lysis buffer (50 mM Tris-HCl, 5 mM EDTA, 150 mM NaCl, 0.5% vol/vol deoxycholic acid, 1% wt/vol NP-40, 1 mM sodium orthovanadate, and 0.2% wt/vol protease inhibitor cocktail; Sigma-Aldrich) for 1 h on ice. The protein concentration of the sample was determined by BioRad DC Protein Assay (Bio-Rad Laboratories Inc., Hercules, CA). Protein extracts were mixed with sample buffer consisting of 60 mM Tris, pH 6.8, 3% (wt/vol) sodium dodecyl sulfate (SDS), 10% (vol/vol) glycerol, 5% (vol/vol) 2-mercaptoethanol, and 0.05% (wt/vol) bromophenol blue, and boiled for 5 min. Protein samples (120 µg of whole-cell lysate) were subjected to SDS-PAGE, and proteins were transferred to nitrocellulose filters (Hybond, Amersham Pharmacia Biotech AB, Uppsala, Sweden). The filters were blocked for 1 h at room temperature with 3% (wt/vol) BSA (bovine serum albumin) in TBS (Tris-buffered saline) with 0.1% Tween-20 and incubated overnight at 4°C with the following primary antibodies: anti-diphosphorylated ERK antibody (Thr183/Tyr185) (Sigma) 1∶10,000, anti-ERK MAPK (BioSource, Rockville, MD) 1∶1000, and anti-p-SAPK/JNK (Thr183/Tyr185), anti-SAPK/JNK, anti-p-p38 (Thr180/Tyr182), anti-p38 and anti *c-jun* (all at 1∶1000; Cell Signaling, Beverly, MA). Primary antibodies were detected by horseradish peroxidase-conjugated secondary antibodies (1∶10,000; Jackson ImmunoResearch Laboratories Inc., West Grove, PA). The targeted protein was revealed by enhanced chemiluminescence (ECL). The membrane was incubated with an ECL solution (Biological Industries) and exposed to ECL film (Eastman Kodak, Rochester, NY) to visualize specifically labeled proteins. The resulting exposed films were then analyzed by densitometry. All experiments were performed at least three times.

### MAPK inhibitor study

Cells were plated at a density of 1×10^6^/100 mm dish (for western blot analysis), or at 5×10^4^/well in a 96-well dish (for XTT viability staining and hemacolor staining) and cultured in serum-free MEM for 16 to 18 h before treatment. Cells were treated with the specified MAPK inhibitors for 2 h, washed and exposed to *A. fumigatus* CF for the indicated times. Western blot analysis was performed as described above. The following MAPK inhibitors were used in this study: (i) FR180204 (50 µM, Calbiochem, San Diego, CA), an ERK1/2 inhibitor [11], (ii) SP600125 (25 µM, Sigma), a selective and reversible inhibitor of JNK, and (iii) SB203580 (25 µM, Calbiochem), a specific inhibitor of p38. For XTT viability assays and microscopy, *A. fumigatus* CF was removed, and the cells were washed twice with PBS and evaluated for cytotoxicity with the XTT assay (see below) or stained with hemacolor. For hemacolor staining, cells were fixed in methanol, stained with hemacolor reagent (Merck, Darmstadt, Germany).

### Azocasein assay for proteolytic activity of CFs

General proteolytic activity was measured by azocasein assay [25], [26]. Azocasein (5 mg/ml; Sigma) was dissolved in 50 mM Tris-HCl (pH 7.5), 0.2 M NaCl, 5 mM CaCl_2_, 0.05% (wt/vol) Triton X-100, and 0.01% (wt/vol) sodium azide. A 400-µl aliquot of this solution was mixed with 100 µl of CF. After overnight growth at 37°C, 150 µl of 20% (vol/vol) trichloroacetic acid was added. After 30 min at room temperature, the tubes were centrifuged at 16,000 *g* for 3 min, and the pellets were discarded. The supernatant was mixed with an equal volume of 1 M NaOH, and absorption of the liberated dye was measured at 436 nm.

### XTT(2,3-bis-[2-methoxy-4-nitro-5-sylfophenyl]-2H-tetrazolium-5-carboxanilide, disodium salt) cell viability assay

A549 cells were grown in 96-well cell-culture plates to 80% confluency. CF was added to the cells following preincubation with the MAPK inhibitors. After incubation, XTT reagent (Biological Industries) was added, and the colorimetric assay was performed as specified by the manufacturer. Absorbance (optical density at 490 nm) was read on an ELISA reader (Spectra MAX 340; Molecular Devices, Sunnyvale CA). Results are representative of three independent experiments performed in triplicate and are expressed as mean ± SD (error bars) of three replicates.

### Flow cytometry analysis of CF-induced apoptosis/necrosis in A549 cells

A549 cells were grown in DMEM with 10% FCS in 6-well plates at a concentration of 5×10^5^/well. The growth medium was replaced with CF and the cells were incubated for various times. The CF was removed and the cells were washed twice with PBS. Untreated WT and protease-deficient CF-treated adherent cells were detached with 0.5% (wt/vol) EDTA (Sigma-Aldrich), washed twice, and labeled with annexin V/propidium iodide [29] (Bender MedSystems, MedSystems Diagnostics GmbH, Vienna, Austria) according to the manufacturer's recommendations. Analysis of apoptosis was performed with a FACSort flow cytometer (Becton, Dickinson and Company, Franklin Lakes, NJ). Results were analyzed with FlowJo software.

### Actin staining

A549 cells were fixed for 30 min at room temperature in PBS containing 3% (vol/vol) paraformaldehyde and permeabilized with 0.5% (wt/vol) Triton X-100 for 3 min. Cells were then incubated for 40 min at room temperature with phalloidin fluorescein isothiocyanate (FITC) (for actin staining) diluted 1∶75 (Molecular Probes, Eugene, ORE), washed three times in PBS and viewed in a Zeiss confocal laser scanning microscope (CLSM 410) equipped with a 25-mW krypton-argon laser and a 10-mW helium-neon laser (543 nm).

### Statistical analysis

All experiments were independently performed at least three times. Unless otherwise stated, a representative experiment is displayed. Error bars denote SDs.

*P*-values were calculated by Student's *t* test or ANOVA. Differences were considered to be statistically significant when *p*<0.05.

## Results

### Microarray analysis

We have previously shown that exposure of A549 cells to *A. fumigatus* germinating conidia or CF results in protease-dependent actin-cytoskeleton destruction, cell rounding, peeling and death [25]. To better understand these processes at the molecular level, we determined changes in gene expression in treated cells by microarray analysis. A549 cells were incubated in the presence of WT or *ΔPrtT* conidia, or WT CF or *ΔPrtT* CF lacking protease activity for 8 h. This time point was selected because we previously demonstrated that at this stage, WT germinating conidia or CF induce partial actin-cytoskeleton depolymerization and cell rounding, without killing the cells [25]. We found minor differences between the transcriptional responses of cells infected with WT or *ΔPrtT* conidia (see Text S1). WT germinating conidia significantly increased the mRNA levels of 115 probes (94 genes) relative to untreated cells. These genes were categorized by the Expander program and included those encoding cytokines (*p* = 1E-9), signal transduction pathways (*p* = 1E-5) and in particular, the MAPK cascade (*p* = 1.17E-10) and transcription factors (*p* = 1.9E-7) (Table 1 and Table S1, all accepted terms are after FDR correction of 0.1). Expander analysis indicated significant enrichment for genes containing NFkB (*p* = 4.25E-6) and MAPK-activated SRF (*p* = 3.86E-5) transcription factor binding sites (Table S2). The mRNA expression level of one gene was significantly decreased by conidial infection relative to untreated cells (NDUFB7, Table S3). Together, results suggested that conidial infection induces a strong protective response in infected A549 cells, characterized by the activation of genes participating in intracellular signaling pathways and the secretion of inflammatory cytokines. Worth noting is the modest number of genes that responded to *A. fumigatus* conidial exposure, unlike the response observed for WT CF which significantly increased the mRNA levels of 357 probes (291 genes) relative to untreated cells (Table S4). These included genes encoding proteins involved in (Table 2) signal transduction and in particular MAPK signaling (MAPKKK cascade *p* = 1.46E-7) and cytoskeletal regulation (*p* = 1.8E-5), transcription factor activity (*p* = 3.19E-8), including negative regulators of transcription. There was significant enrichment for genes containing E2-F1 (*p* = 9.35E-7), AP-2α (*p* = 1.15E-5) and Sp1 (*p* = 3.21E-6) binding sites (Table S5). These transcription factors have been shown to induce cell-cycle arrest and apoptosis [30], [31].

**Table 1:** Genes upregulated in response to wild-type conidial infection.

GENE CATEGORIES	Genes Up regulated by wild-type conidial infection
Cytokine signaling and inflammation (GO:0005125)	AREG, MCP1 (CCL2), CCL20, CXCL1, CXCL2, CXCL3, IL6, IL8, LIF, PTGS2, VEGF
Signal Transduction (GO:0007166)	ADM, AKAP12, BIRC3, CTGF, DUSP1 ^MK^, DUSP4 ^MK^, DUSP5 ^MK^, DUSP6 ^ MK^, DUSP8 ^MK^, FST, GEM, GPRC5A, PLAUR, RGS2, SOCS5, SOCS6, SPRY4 ^ MK^, STC1,TRIB1 ^MK^
Transcription factor activity (GO:0003700)	ATF3, BCL3, BHLHE40, CBFB, EGR1, FOS, FOSL1, FOXO1, JUN, JUNB, KLF5, KLF6 ^C^, MAFF, NFkBIA, NAB1, NFIL3, NRG1 NR4A1, NR4A2, NR4A3, SERTAD2, TNFAIP3

**Table 2:** Genes upregulated in response to WT CF treatment.

GENE CATEGORIES	A549 genes upregulated in response to WT CF treatment
Cytokine signaling (GO:0005125)	CCL2, HRH1, IL8, IL1RL1, IL6R, IL27RA
Signaling PW (GO:0007242)	ARHGDIA, BAIAP2 ^C^, BDNF^MK^, CAV1 ^MK^, CCND1, CCNE2, DCBLD2, DUSP6 ^MK^, DVL1, EPHA2, EREG, E2R^C^, F2R, FAT1^C^, FAS, FGF2, FGFBP1, FGFR1^MK^, FST, FSTL3, GTSE1 ^MK^, ITGA3^C^, KRTI5 ^C^, MAP3K2 ^MK^, MAP4K5 ^MK^, MAPKAP ^MK^, NRG1, NRP2, OXTR, PAK2^C^, PLAUR, PTHLH, PLCL2, P2RY2, RGS20, RAB35 ^C^, RALA ^C^, RAP2A ^C^, RHOB ^C^, RHOF ^C^, SERPINE1, SFN, SGK1, SFRP1, SMURF2, SOCS2, SOCS5, SOD2, SOS1, TAOK ^MK^, TGFA, TGFB2, THBD, TMX1, TNFRSI9, TRIB1 ^MK^, VEGCF
Transcription factor activity (GO:0003700)	ARNTL2, CNOT3, CSRNP2, ETS, ETS2, FOSL1, FOSL2, JUN, KLF4, KLF5, KLF6^C^, LMO4, MAFF, MDFIC, MSX1, MYBL1, NFKB2^I^, NFKB1A, NFKBIE, NKX3-1, NPAS2, NR5A2, PPARG, RELB, SBNO2, SOX9, TBX3, WWTR1, ZBTB1, ZFP36L1, ZNF174, ZNF557
Response to unfolded protein (GO:0006986)	DNAJB6, HSPA1A, HSPH1

Underlined  =  negative regulator.

MK  =  MAPK pathway, GO:0000165.

C  =  cytoskeleton, GO:0005856.

HSP  =  heat-shock protein.

CF significantly decreased the mRNA levels of 396 probes (324 genes) relative to untreated cells (Table S6). These included genes encoding proteins involved in amino acid nitrogen and lipid metabolism processes (cellular amino acid and derivative metabolic process, *p* = 4.45E-21, lipid metabolic process, *p* = 9.38E-9 and nitrogen compound metabolic process, *p* = 6.92E-10), suggesting a general shutdown of energy-intensive metabolic processes (Table 3).

**Table 3:** Genes downregulated in response to WT CF treatment.

GENE CATEGORIES	Genes downregulated in response to WT CF treatment
Amino acid and derivative metabolism (GO:0006519)	AARS, AKR1C1, CARS, EPRS, GARS, GCLC, GCLM, GSTA1, IARS, IDH1, MARS, MTHFD2, PCK2, SARS, SLC3A1, SLC7A7, SLC23A2, TARS, YARS
Nitrogen compound metabolism (GO:0006807)	ALDH6A1, ASL, ASMTL, ASNS, ASS1, AUH, BCAT1, CBS, CORO2A, CPS1, HGD, HMOX1, LGSN, MAOA, NAMPT, PHGDH, PSAT1, PSPH, PYCR1
Lipid metabolism (GO:0006629)	ABCA1, ABCG1, ACACA, ACSM3, ALG13, BDH2, CYP4F3, DHRS3, GDPD5, HMGCS2, PLA2G6, PLCXD1, PLD1, SCD, SCD5, SREBF1, VLDLR

A comparison of the effects of WT CF and *ΔPrtT* CF on A549 gene expression showed that the former significantly increased the mRNA levels of 259 probes (226 genes) relative to the latter (Table S7). These included genes encoding proteins involved in cell-cycle control (cell cycle, *p* = 3.66E-39), and in particular regulation of microtubules (microtubule cytoskeleton, *p* = 1.86E-20) and the unfolded protein response (UPR) (response to unfolded protein, *p* = 4.65E-6, Table 4), which suggests that the WT-CF-treated cells could be modulating cell cycling and protein synthesis and folding in response to protease activity or other *PrtT*-dependent secreted proteins in the WT filtrate. There was significant enrichment in genes containing binding elements for the transcription factor NF-Y (*p* = 4.79E-19, 40/75 genes, Table 4, underlined). The mRNA levels of 517 probes (473 genes) were significantly reduced in WT-CF-treated cells relative to those treated with *ΔPrtT* CF (Table S8). These included genes encoding proteins involved in signal transduction (regulation of cell communication, *p* = 4.35E-15 and regulation of small GTPase-mediated signal transduction, *p* = 1.12E-12) and in particular vesicle trafficking (*p* = 1.84E-6), transporter activity (*p* = 1.81E-5) and actin-cytoskeleton dynamics (cytoskeleton organization, *p* = 4.95E-7), suggesting that these functions are impaired by the protease activity in the WT CF. There was significant enrichment in genes containing binding elements for the transcription factor MOVO-B (*p* = 1.12E-5, Table 5, underlined) which is involved in promoting angiogenesis [32].

**Table 4:** Genes upregulated in response to WT CF vs. ΔPrtT CF.

GENE CATEGORIES	Genes upregulated in response to WT CF vs. ΔPrtT CF
Cell cycle (GO:0007049)	ANLN, ASPM, AURKA ^C^, BARD1, BIRC5, BUB1, BUB1B ^C^, CCNA2, CCNB1, CCNB2, CDC2, CDC25, CDCA2, CDCA3, CDCA5, CDK6, CDKN3, CENPF, CEP55, CIT, CHTF8, CKAP2, CKAP5 ^C^, DBF4B, DCDC2, DLGAP5, DUSP6, ECT2, EDN1, ENC1, EPB41L2, ESCO2, GADD45A, GAS2L3, GINS1, GTSE1 ^C^, INCENP, KIF11 ^C^, KIFF14 ^C^, KIF23 ^C^, KIF2C ^C^, KIF4A ^C^, KIF18B ^C^, KIF20A ^C^, KIFC1 ^C^, LMNB1, NCAPG, NGAG2, NCAPH, NUF2, NUSAP1 ^C^, PLK2, PBK, PRC1 ^C^, PTTG1, RACGAP1, RGS2, SPAG5 ^C^, STMN1 ^C^, TACC3, TPX2, TXNIP, UBE2C ^C^, ZWILCH
Unfolded protein response (GO:0006986)	ASF1B, HSPH1,HSPA1A, HSPA1B, HSPA4L, HSP90AA1, DNAJA1/HSP40, DNAJB1/HSP40, HSPA6/HSP70B, DNAJB4/HSP40, SERPINH1

Underlined: genes containing NF-Y promoter binding sites.

C  =  Cytoskeleton (microtubule) GO:0015630.

**Table 5:** Genes downregulated in response to WT CF vs. ΔPrtT CF.

GENE CATEGORIES	Genes downregulated in response to WT CF vs. ΔPrtT CF
Signal transduction and cell communication (GO:0010646)	ABCA1^ T^, ABLIM3^ c^, ABR, ACVR1, AGAP6, AP4M1^ T^, ARAF, ARAP3 ^ c^, ARNT, ARFGAP3 ^ c^, ARHGAP5 ^ c^,ARHGAP32^ c^, ARHGEF3 ^ c^, ARL4D^ T^, BAIAP2L1^ c^, BCAR3, BDKRB1, BDKRB2, BMP6, CLN8^ T^, CORO2B^ c^, CTNNB1^ c^, CYTH2^ c^, DAPK2, DNMPB^ c^, DUSP10, EXOC2^ T^, FHL3^ c^, F2R, F2RL1, GARNL3, GPR56, GREM1, GRK5, GSN^ c^, HOOK2^ c^, HTR1D, IL11, IL4R, IL17RA, IRAK2, ITGA2, ITGA4, ITGA5, ITGA11, KCNAB2 ^ T^, KCNN4^T^, KCTD21 ^ T^, LIF, LITAF, LRP4^ T^, LRRN2, LYST^ T^, MLPH^ c^, MAP1S^ c^, MAP3K14, MAPK7, MAP7 ^ c^, MRAS, MRC2 ^ T^, MTSS1^ c^, MYO9B^ c^, NHEDC2^T^, NPAS, NPC1^T^ NPR2, ORC6L, PACS1^ T^, PDGFA, PITPNC1 ^T^, PITPNM^ T^, PLAUR, PLA2R1,PLCB1, PLCG1, PLEK2^ c^, PLEKHG2, PLEKHM1, PPARD, PREX1 ^ c^, PSD3, PTPRK, PYARD, RAB31^ T^, RAB43^ T^, RAB7L1^ T^, RALGAPA2, RAP1GAP ^ T^, RAP1GAP2 ^ T^, RASA2, RASA3, RASA4, RASEF^ T^, RASGRP1, RASGRP3, RHOBTB2, RHOU^ c^, RIPK1, RND1^ c^, SFRP, SH2B2, SH3KBP1^ c^, SIPA1L2, SLC14A1^T^, SLC17A5 ^ T^, SLC19A3^T^, SLC20A1 ^ T^, SLC22A23 ^ T^, SLC2A13^ T^, SLC6A16^ T^, SPHK1, SPSB1, SQSTM1, STK38L, STX1A^ T^, STX4 ^ T^, SYNRG^ T^, SYT13^ T^, TBC1D25, TBC1D3B, TCIRG1^ T^, TGFB1, TGFB2, TGFBR1, TMOD1^ c^ TNFRSF1A, TOM1^ T^, ULK1, VEGFA, VPS41^ T^, WIPF1C^ c^

C  =  cytoskeleton, GO:0007010.

T  =  transport, GO:0006810.

Underlined: genes containing MOVO-B promoter binding site.

**Figure 1:**
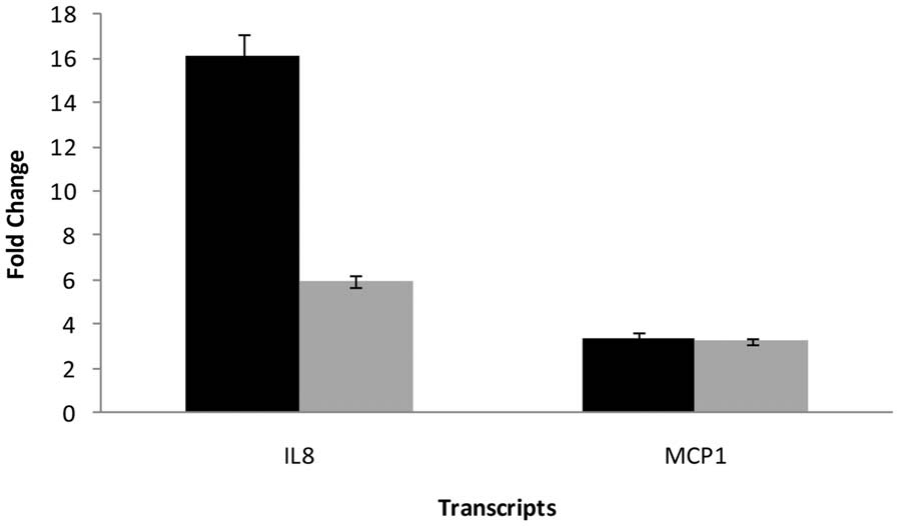
Validation of changes in cytokine gene expression levels. A549 cells were grown with either WT conidia or WT-CF for 8 h. Control cells had no added fungi or CF. RT-PCR was used for semi-quantitative analysis of IL-8 and MCP1 transcripts. IL8 levels showed a 16-fold rise in response to conidial infection (black) and a 6-fold increase in response to WT CF (grey). Conidial infection and WT-CF treatment activated MCP1 transcription to similar levels (approximately 3-fold) as measured by the microarrays. Data are presented as mean ± SD.

We independently validated the microarray results for two key cytokines, IL8 and MCP1, by RT-PCR analysis (Fig. 1). Microarray analysis showed a 103-fold increase in IL8 mRNA levels in response to conidial infection, and a 4-fold increase in response to WT CF. A parallel increase was seen by RT-PCR analysis. Conidial infection and WT-CF treatment increased MCP1 mRNA to 10 and 10.9-fold respectively as measured by the microarrays. A similar, though attenuated response was seen by RT-PCR analysis. It is important to note that our RT-PCR microarray validation analyzed only the expression of the two key cytokines IL8 and MCP1 and did not include genes induced specifically by WT-CF compared to *ΔPrtT* CF.

### *A. fumigatus* CF activates ERK and JNK signaling in A549 cells in a protease-dependent manner

**Figure 2:**
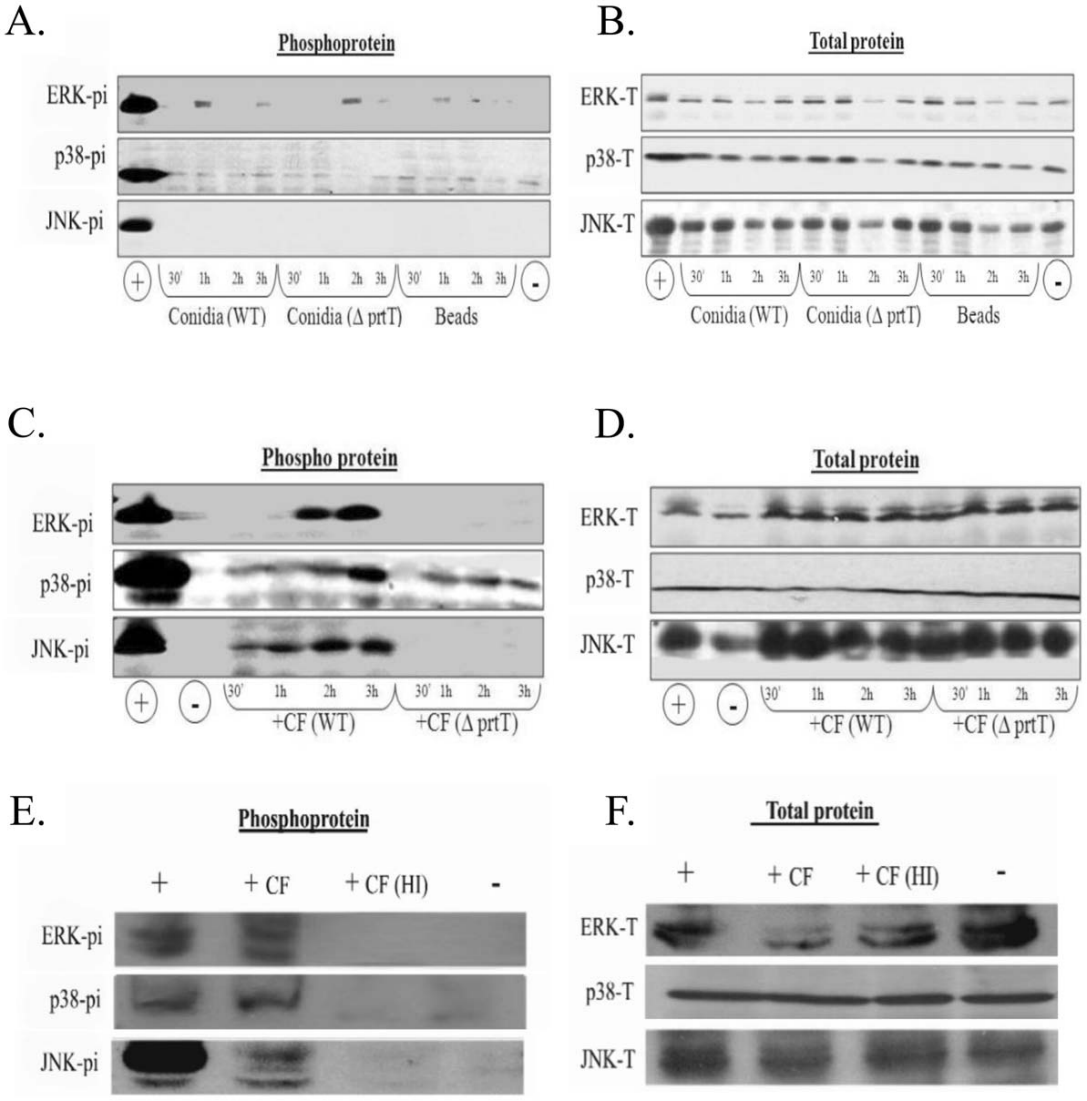
Phosphorylation of ERK1/2, JNK, and p38 is activated by WT CF. A549 cells were treated for 30 min, 1 h, 2 h or 3 h with (A–B) WT or *ΔPrtT* conidia, or inert polystyrene beads, (C–D) WT CF or *ΔPrtT* CF (lacking protease activity) or for 3 h with (E-F) WT-CF and heat-inactivated (HI) CF lacking protease activity. We used hydrogen peroxide (H_2_O_2_)-treated cells as a positive control (+), and carrier-treated cells as a negative control (−). Phosphorylation of ERK1/2, JNK, and p38 was monitored by western blotting of whole-cell extracts with specific anti-p-ERK (Thr183/Tyr185), anti-p-SAPK/JNK (Thr183/Tyr185), and anti-p-p38 (Thr180/Tyr182) MAPK antibodies (A, C, E). Total MAPK levels were monitored by ERK1/2, JNK and p38-specific antibodies (B, D, F).

Based on the results of the microarray analysis, we hypothesized that *A. fumigatus* germinating conidia or CF activate MAPK signaling in A549 cells. To test this hypothesis, A549 cells were incubated in the presence of WT and *ΔPrtT* conidia and CFs, harvested after 0.5, 1, 2 and 3 h and analyzed by western blot with phospho-specific and protein-specific anti-ERK1/2, p38 and JNK antibodies. These time points were selected because we reasoned that kinase phosphorylation occurs rapidly, before the transcriptional and morphological changes seen after 8 h of exposure. As a control for conidial infection, cells were incubated in the presence of 2- to 4-µm diameter polystyrene beads. The results indicated that WT and *ΔPrtT* conidia activate some residual ERK signaling in comparison to the polystyrene beads (Fig. 2A–B). Conidial infection for up to 6 h (during which time hyphal growth had begun) did not result in significant MAPK activation (data not shown). In contrast, WT *A. fumigatus* CF induced the phosphorylation of ERK1/2, p38 and JNK after 1 to 2 h of incubation. Interestingly, *ΔPrtT*-derived CF did not induce ERK1/2 or JNK phosphorylation and only partially activated p38 phosphorylation (Fig. 2C–D). Heat- inactivation of WT-CF proteolytic activity blocked its ability to activate ERK1/2 or JNK phosphorylation (Fig. 2E-F). This suggests that in CF-treated A549 cells, ERK and JNK phosphorylation occurs in response to the proteolytic activity of secreted fungal proteases or possibly to the activity of additional proteins secreted in a *PrtT*-dependent manner.

### Inhibition of ERK or JNK signaling partially protects A549 cells from CF-induced cell peeling and loss of viability

We reasoned that CF-induced phosphorylation of ERK, JNK and p38 kinases in A549 cells plays an important role in orchestrating later changes in cell morphology and viability. We therefore preincubated the cells in the presence of ERK1/2, JNK or p38-specific inhibitors, followed by addition of CF for 12 h.

**Figure 3:**
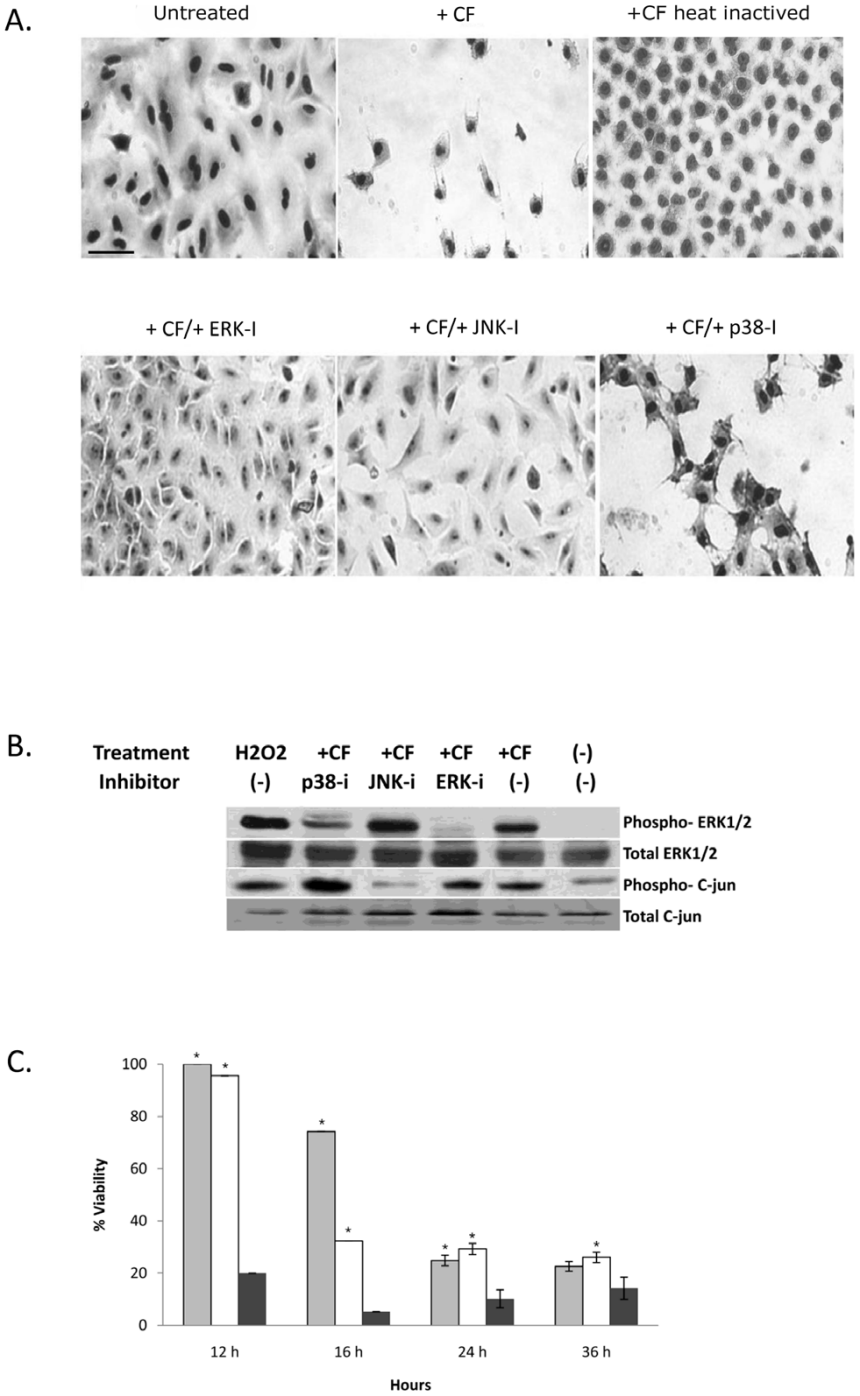
MAPK inhibitors protect A549 lung epithelial cells from loss of viability induced by CF treatment. (A) ERK1/2 and JNK inhibitors block CF-induced cell peeling. A549 cells pretreated for 2 h with 50 µM ERK-I (FR180204), 25 µM JNK-I (SP600125) or 25 µM p38-I (SB203580) washed, incubated for 12 h in the presence of WT CF, stained with hemacolor and analyzed by microscopy (bar  = 100 µm for all images). Cells pretreated with ERK-I or JNK-I remained attached to the plate and retained their elongated shape. Cells treated with heat-inactivated WT CF lacking protease activity retained their normal shape (B) Verification of ERK1/2 and JNK inhibitor specificity. A549 cells pretreated with ERK-I, JNK-I or p38-I were washed, incubated with WT CF for 3 h, lysed and analyzed by western blot with ERK1/2 and c-Jun-specific antibodies. (C) ERK1/2 and JNK inhibitors block CF-induced cell death. A549 cells pretreated with ERK-I (grey), JNK-I (unshaded) or a carrier control (black) were washed and incubated with WT CF for 12, 16, 24 or 36 h. Cell viability was determined by the XTT colorimetric assay which measures mitochondrial metabolic activity. Shown are the means standard deviations (error bars) for three independent experiments. *, P value of <0.05 for cell viability following inhibitor treatment relative to untreated cells at the same time-point.

A549 cells were subsequently analyzed for changes in morphology, ERK1/2/JNK/p38 phosphorylation and viability. Heat- inactivation of WT-CF proteolytic activity blocked its ability to peel the cells. Inhibition of ERK and JNK signaling partially blocked CF-induced A549 cell peeling, whereas inhibition of p38 kinase activity did not (Fig. 3A). Western blot analysis showed that the ERK1/2 and JNK inhibitors specifically inhibited CF-induced ERK1/2 autophosphorylation and *c-jun* (a direct downstream target phosphorylated by JNK) phosphorylation, respectively (Fig. 3B). CF-induced reduction in A549 cell viability, as measured by the reduction in XTT, was markedly delayed (for up to 16 h) following incubation of the cells in the presence of the ERK1/2- and JNK-specific inhibitors (Fig. 3C). At later time points (24 and 36 h incubation in the presence of CF), cell viability was strongly reduced despite the pretreatment with the inhibitors.

### Inhibition of ERK or JNK signaling partially protects A549 cells from CF-induced actin-fiber depolymerization and necrosis

**Figure 4:**
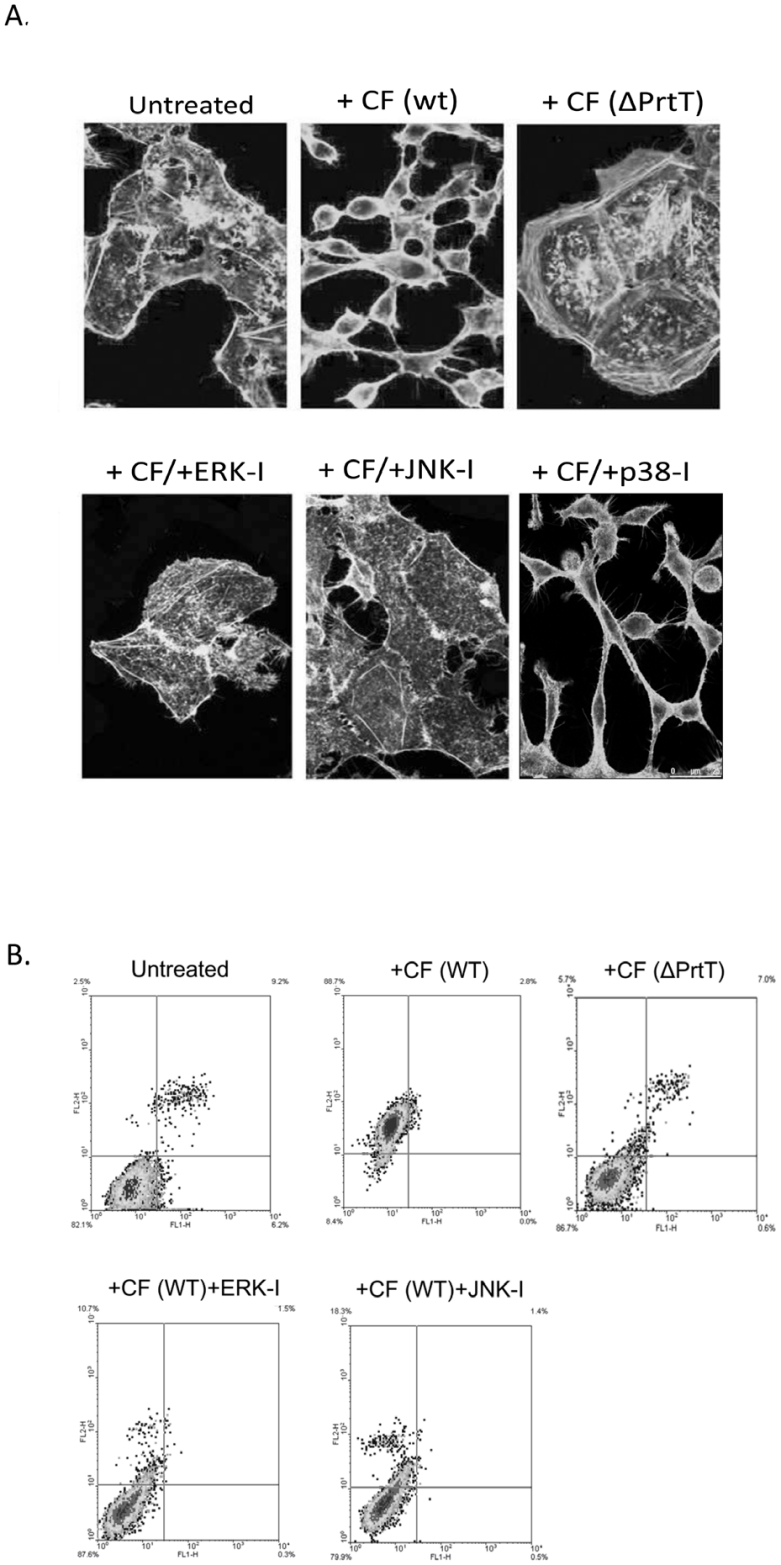
ERK1/2 and JNK inhibitors block CF-induced actin-fiber depolymerization and necrotic death. A549 cells pretreated with ERK-I, JNK-I or p38-I were washed and incubated with WT CF for 8 h. Cells treated with *ΔPrtT* CF (lacking protease activity) or carrier (untreated) served as negative controls. Cells were (A) stained for actin with phalloidin-FITC and analyzed by confocal microscopy. Cells treated with ERK-I or JNK-I retained their flattened shape and network of actin fibers. (bar  = 20 µm for all images) whereas cells treated with p38-I did not (B) assayed for apoptosis/necrosis by flow cytometry following annexin V (x-axis)/PI staining (y-axis) of the cells. WT CF induces protease-dependent cellular necrosis which is blocked by ERK1/2 and JNK inhibitors. Upper panel: necrosis is induced in A549 cells after 12 h of incubation in the presence of WT CF but not *ΔPrtT* CF. Lower panel: ERK-I and JNK-I inhibit the induction of necrosis in A549 cells under WT CF treatment. Experiments were repeated three times with similar results. A representative experiment is shown.

We previously showed that the proteolytic activity of CF induces rapid (2–8 h) depolymerization of the actin cytoskeleton in treated A549 cells, leading to subsequent cell detachment and death [25]. Here, we reasoned that the ERK1/2 and JNK inhibitors might protect the cells by inhibiting these events. A549 cells were preincubated for 2 h with control medium or medium containing JNK or ERK inhibitors, washed repeatedly, and further incubated for 8 h in the presence of WT or *ΔPrtT*-derived CF (Fig. 4A). Actin fibers were visualized by phalloidin FITC staining and fluorescence microscopy. Addition of ERK or JNK inhibitor alone did not affect actin-fiber polymerization (data not shown). WT CF caused actin-fiber depolymerization whereas *ΔPrtT* CF did not. Interestingly, treatment with JNK or ERK1/2 inhibitors blocked WT-CF-induced actin-fiber depolymerization and cell rounding, suggesting that these processes are dependent on JNK or ERK1/2 activity.

Previous studies have indicated that CF-treated A549 cells die mainly by necrosis, with some cells initiating apoptosis but ultimately also undergoing necrosis [23], [25]. To determine whether the JNK or ERK1/2 inhibitors delay CF-induced A549 necrosis or apoptosis, cells were pretreated with the inhibitors as described above and incubated for 12 h in CF. The cells were harvested, stained with PI (necrosis) and annexin-V-FITC (apoptosis) and analyzed by flow cytometry. Results showed that WT CF and *ΔPrtT* CF induce necrotic death in 89% and 6% of the cells, respectively, suggesting that necrosis is primarily mediated by *PrtT*-regulated secreted proteins, possibly including, fungal proteases (Fig 4B). Interestingly, preincubation of the cells with ERK1/2- or JNK-specific inhibitors inhibited necrosis to 11% and 18% of the cells, respectively (Fig. 4B). These results imply that CF-induced cell necrosis is dependent on both *PrtT*-regulated secreted proteins and the subsequently activated JNK or ERK1/2 signaling in the exposed cells.

## Discussion

We describe the transcriptional response of cultured A549 lung epithelial cells to infection by *A. fumigatus* germinating conidia or treatment with CF. We show that CF induces MAPK (ERK1/2, JNK, p38) phosphorylation in treated A549 cells in a *PrtT*-dependent manner. *PrtT* is a key *A. fumigatus* transcription factor regulating the expression of secreted proteases [26], [27]. Inhibition of ERK1/2 or JNK activity partially protected the cells from CF-induced damage, suggesting that these kinases are an important component in the induction of cell peeling, loss of actin fibers and necrosis.

### The transcriptional response of A549 lung epithelial cells to *A. fumigatus* conidial infection or exposure to CF

We chose to compare both conidial and CF models of cell exposure because germinating conidia represent the earliest phase of infection, whereas CF may partly recapitulate aspects of late infection, such as the secretion of enzymes and toxins by a developed hyphal mass. Late infection cannot be reproduced by infecting cells for an extended period with germinating conidia, because they form a hyphal mass which cannot be separated from the cells.

A549 cells responded to conidial infection by activating genes characteristic of a prototypical “host core alarm response' previously shown to be induced in epithelial cells infected by viruses or bacteria [33], [34]. This includes the activation of genes encoding cytokines, initiating inflammation, and activating the NFkB and MAPK/AP-1 (jun/fos) signaling pathways, as well as genes that limit the immune response such as NFkBIA, TNFAIP3 and the DUSP-MAPK-phosphatases. In marked contrast, WT-CF treatment of A549 cells induced a transcriptional response characterized by a reduction in the mRNA levels of genes involved in amino acid/lipid metabolism and protein translation. This suggests that CF treatment damages the cells, shutting down the transcription of genes involved in vital cell functions. WT CF increased the mRNA levels of genes involved in signal transduction and in particular the MAPK pathway, as well as genes involved in regulating cytoskeletal changes. Indeed, both processes are strongly affected in CF-treated cells (see proceeding sections).

Interestingly, conidial infection activated more cytokine-encoding genes than the WT CF treatment. This could reflect the fact that the conidia physically interact with the infected cells. This interaction involves binding of conidial surface polysaccharides by cellular pattern recognition receptors, activating a strong cytokine response [12], [35].

A comparison of the A549 cells' responses to treatment with WT CF and *ΔPrtT* CF showed several differences: WT CF, unlike *ΔPrtT* CF, activated the expression of genes involved in cell-cycle control and the UPR. The UPR is activated in response to the accumulation of unfolded or misfolded proteins in the lumen of the endoplasmic reticulum, and involves the arrest of protein synthesis and the cell cycle and upregulation of proteins involved in chaperoning misfolded proteins and protein folding [36]. Activation of the UPR might be attributable to the activity of secreted proteins, including proteases found in the WT CF and not in the *ΔPrtT* CF.

### *PrtT*-regulated proteins secreted by *A. fumigatus* induce JNK, ERK1/2 and p38 phosphorylation in exposed A549 cells

*A. fumigatus* WT CF rapidly induced phosphorylation/activation of JNK, ERK1/2 and p38 in A549 cells, whereas infection of the cells with germinating conidia did not. CF-induced phosphorylation of ERK1/2 and JNK was dependent on the presence of *PrtT*-regulated secreted proteins, including proteases, as it did not occur when heat inactivated WT-CF or CF from a *PrtT*-deficient strain of *A. fumigatus* was added to the cells.

The MAPKs JNK, ERK1/2 and p38 are activated by phosphorylation induced by various mitogens and cell stressors, including osmotic stress, heat shock and proinflammatory cytokines. They regulate diverse cellular activities, such as gene expression, mitosis, differentiation, cytoskeletal rearrangement, proliferation and apoptosis [37]. MAPK activation also occurs in cells infected by various pathogens, including viruses [38], [39], [40], bacteria [41], [42], [43] and some fungi [9], [44], [45], [46], [47], [48]. Interestingly, specific MAPK inhibitors can, in some cases, block the entry or proliferation of the infecting virus or bacteria in infected cells [39], [41], [43]. In contrast, inhibition of ERK signaling in macrophages or neutrophils infected with *Candida albicans* has been shown to impair these cells' ability to migrate toward and kill the fungus [46], [48], [49]. Relatively little is known about the role of host-cell MAPKs in response to *A. fumigatus* infection. Infection of the human bronchial epithelial cell line BEAS-2B with germinating *A. fumigatus* conidia resulted in phosphorylation of both ERK1/2 and p38 and subsequent release of IL8 [9]. In contrast, in this work, MAPK phosphorylation was not specifically induced by germinating *A. fumigatus* conidia, reflecting a possible difference in the response of the A549 human alveolar type II cell line used in this study and that of the BEAS-2B line [9]. Here, we show for the first time that *A. fumigatus* CF induces *PrtT*-dependent JNK, ERK1/2 and p38 phosphorylation in treated A549 cells. Since deletion of *PrtT* results primarily in the loss of secreted protease activity, it is likely that this activity may be responsible for the subsequent MAPK activation in the treated A549 cells [49]. Secreted proteases have been shown to cleave and activate A549 cell-surface receptors, subsequently activating downstream signaling elements, including the MAPK cascade. For example, proteolytic activation of the PAR-2 receptor in lung epithelial cells has been shown to result in a strong inflammatory response characterized by the release of proinflammatory cytokines, prostaglandins and leukocyte recruitment [50]. A549 cells express PAR-2, and its proteolytic activation by the dust mite serine protease allergen Der p 3 results in the activation/phosphorylation of ERK1/2 and release of IL8. Interestingly, this response can be blocked by the addition of ERK1/2 inhibitors [51], [52].

### Inhibition of JNK or ERK1/2 activity in CF-exposed A549 cells partially protects them from damage and death induced by *A. fumigatus* CF

We show that addition of JNK or ERK1/2 inhibitors, but not p38 inhibitors, to A549 cells treated with WT CF markedly delays (i) loss of cell adhesion and viability as measured microscopically and by the XTT cell viability assay, [11] depolymerization of actin fibers and subsequent cell rounding as measured by actin fluorescence staining, and (iii) necrotic cell death as quantified by annexin V/PI staining and flow cytometry.

Treatment with these inhibitors increased the survival time of treated cells from 6–12 h (untreated) to 16–24 h (treated with JNK or ERK1/2 inhibitors). Previous work has shown that ERK1/2 and p38 inhibitors can block the inflammatory response of human bronchial epithelial cells infected by germinating *A. fumigatus* conidia [9], but to the best of our knowledge, this is the first time that MAPK inhibitors have been shown to produce an overall protective response in cells treated with fungal CF. Our results suggest that some of the damage occurring in the cells in response to CF is self-inflicted: *PrtT*-regulated secreted proteins, likely including proteases induce A549 cell MAPK signaling which promotes pathways that actively lead to loss of actin fibers, cell rounding and ultimately, necrotic death. We previously demonstrated similar partial protection of A549 cells treated with WT CF in the presence of protease inhibitors [25]. In both cases, only partial protection suggests that in addition to proteases, other fungal-secreted components, such as gliotoxin, are involved in this process. The partial protection we observed *in vitro* may also explain why the *A. fumigatus PrtT* deletion mutant exhibits apparently normal virulence in infected mice [26], [27]: since invasive aspergillosis is a multifactorial infection, lack of protease activity in this fungus may be masked by additional virulence mechanisms. Additionally the mouse model uses a very large conidial inoculum for infection and may not be sufficiently subtle to detect small differences in virulence. Our results suggest, however, a possible therapeutic approach in which *A. fumigatus* infection is treated by a combination of antifungals and MAPK inhibitors which can modulate an excessive host inflammatory response [37], [53]. Indeed, tissue necrosis in steroid-treated non-neutropenic IPA is primarily mediated by inflammatory neutrophilic and monocytic infiltrates [54].

In summary, we carried out an initial characterization of the signaling and transcriptional events occurring in a lung alveolar cell line exposed to *A. fumigatus* germinating conidia and CF and identified the important role of MAPK signaling in the cellular response. Future work will focus on the identification of the signal-transduction pathways mediating MAPK activation in the CF-treated cells. We will also test the applicability of treating steroid-dependent IPA in a mouse model with a combination of antifungals and MAPK inhibitors.

## Supporting Information

Table S1 Genes upregulated in response to wild-type conidial infection relative to untreated cells.
(XLS)

Table S2 Genes upregulated in response to wild-type conidial treatment and containing NFkB or SRF transcription factor binding sites.
(DOC)

Table S3 Genes downregulated in response to wild-type conidial infection relative to untreated cells.
(XLS)

Table S4 Genes upregulated in response to wild-type CF treatment relative to untreated cells.
(XLS)

Table S5 Genes upregulated in response to wild-type CF treatment and containing AP-2, Sp-1 or E2F transcription factor binding sites.
(DOC)

Table S6 Genes downregulated in response to wild-type CF treatment relative to untreated cells.
(XLS)

Table S7 Genes upregulated in response to wild-type CF relative to ΔPrtT CF.
(XLS)

Table S8 Genes downregulated in response to wild-type CF relative to ΔPrtT CF.
(XLS)

Text S1 (DOC)
