# Supplementary material for: PrtT-Regulated Proteins Secreted by Aspergillus fumigatus Activate MAPK Signaling in Exposed A549 Lung Cells Leading to Necrotic Cell Death
Source: PLoS One. 2011 Mar 11;6(3):e17509. doi: 10.1371/journal.pone.0017509 (PMC3055868; doi:10.1371/journal.pone.0017509)
Supplement: Table S2 — Genes upregulated in response to wild-type conidial treatment and containing NFkB or SRF transcription factor binding sites. (DOC) [file pone.0017509.s002.doc]

| TF enrichment | Conidial infection |
| --- | --- |
| NFkB-regulated genes | IER3, CXCL1, PTGS2, FERMT2, CXCL2, CXCL3, BIRC3, CCL20, TOB2, GEM, NR4A2, TNFAIP3, MAPK6, CCNL1, NFKBIA, IL8 |
| SRF-regulated genes | ADM, EGR1, FAM60A, FERMT2, FOS, DUSP5, AKAP12, FOSL1, IL6, PLAUR, MCL1, INSIG2 |
