# Supplementary material for: PrtT-Regulated Proteins Secreted by Aspergillus fumigatus Activate MAPK Signaling in Exposed A549 Lung Cells Leading to Necrotic Cell Death
Source: PLoS One. 2011 Mar 11;6(3):e17509. doi: 10.1371/journal.pone.0017509 (PMC3055868; doi:10.1371/journal.pone.0017509)
Supplement: Table S5 — Genes upregulated in response to wild-type CF treatment and containing AP-2, Sp-1 or E2F transcription factor binding sites. (DOC) [file pone.0017509.s005.doc]

| TF enrichment | WT CF treatment |
| --- | --- |
| AP-2-regulated genes  (p-value 1.15E-5) | SENP3,EML1,HOMER3,F2R,COL7A1,TAOK2,KLF6,SFRP1,NME6,PPP3CC, VASH2,MBD4,ITGA3,SBNO2,COL5A1,SOCS5,RGS20,POLRMT,GPR172A, SYNJ2,QKI,ZFP36L1,TUBB2A,COL4A1,PCGF3,COL4A2,LMO4,PLXNA1, TSPAN5,HIP1,KIAA1199,CHMP2B,RAB11FIP1,EIF5,ADAM19,P2RY2,ZNF131,TIMP3,MICAL2,SRRT,NOP16,B3GNT2,TUBB2B,GPR125,FSTL3,TRIB1,TGFB2,DCAF17,RBPJ,CSRNP2,VEGFC,AHNAK,BDNF,PODXL,DUSP5,PXN,SEC24A,FEM1B,CCND1,ALDH1A3,ROD1,PDSS1,RALA,DEGS1,H2AFX,SERPINE1,EZR,ZBED4,ARHGDIA,LHFP,KIF3A,SLC26A2,TTPAL,CLN8,MREG,ZYX,MICALL1,FAT1,SOCS2,FGF2,SEC14L1,CCDC41,ABHD2,TBX3,EPHB2,SOX9,PLCL2,SOD2,TGFA,WWTR1,DVL1,MAP4K5,TFPI2,NRP2,MAP7D1,PALM2,AKAP2,SGK1,SOLH,ETS2,C8orf55,PVRL3,SMURF2,EXT1, EGFR,FOSL2,CD59,ZBTB1,STK10,CD44,FST,ITGA6,CYP1B1,CPM,IL22RA1,SGMS1,RHOF,GTF3C4,HERC4,NPAS2,PMEPA1,IL6R,ARNTL2,EPHA2,C11orf68,ANKLE2,CAV2,CHST11,METAP2,MSX1,IL27RA |
| Sp1-regulated genes (p-value 3.21E-6) | AHNAK,AKAP12,ARFGEF2,ARHGDIA,ATP6V0A2,B3GNT2,BAIAP2,BDNF,BICD2,C11orf68,C17orf91,C8orf55,CA8,CAV1,CCL2,CCNE2,CD44,CD59,CHST11,CLN8,CNN3,CNOT3,COL4A1,COL4A2,COL5A1,COL7A1,CPM,CST2,CYP1B1,DCAF17,DCBLD2,DKK1,DNAJB6,DUSP5,DUSP6,DVL1,EFHD2,EGFR,EHD1,EIF5,ELL2,EML1,EXT1,F2R,F2RL1,FBXL5,FEM1B,FEM1C,FERMT2,FGF2,FGFR1,FOSL1,FOSL2,FST,GABRA5,GAS2L1,GLS,GPR172A,HIP1,HSPH1,INO80B,ITGA3,ITGA6,JUN,KIAA1199,KIF16B,KIF3A,KLF4,KLF5,LMO4,LSR,LYST,MAFF,MAP3K14,MAPKAPK2,MBD4,MCM10,MFSD9,MICAL2,MICALL1,MRPS12,MSX1,NFKB2,NFKBIA,NFYA,NMT2,NOP16,NPAS2,NRG1,NRP2,P2RY2,PAK2,PALM2,AKAP2,PANX1,PCGF3,PHLDA1,PHLDA2,PMEPA1,POLRMT,PPIF,PPP3R1,PTRF,PVRL3,PXN,QRSL1,RAB11FIP1,RAB35,RAB3B,RALA,RAP2A,RBPJ,RGS20,RHOB,RHOF,SBNO2,SEC14L1,SEMA3C,SENP3,SERPINE1,SFN,SGK1,SGMS1,SLC26A2,SLC39A7,SLC7A6,SMURF2,SOCS2,SOCS5,SOD2,SOLH,SOS1,SOX4,SOX9,SPAG9,SPTLC2,SRD5A1,SS18,SYNJ2,TAOK2,TFPI2,TGFA,TGFBR3,TLE4,TNFRSF12A,TNFRSF9,TRIO,TSC22D2,TSPAN5,TTPAL,TUBB2A,TUBB2B, TUBD1,UBE2Z,VANGL1,VASH2,VEGFC,WWTR1,ZBED4,ZFP36L1,ZNF131,ZNF174,ZYX |
| E2F-regulated genes (p-value 9.35E-7) | ACTR5,ADAM19,AKAP12,ALDH1A3,ANKLE2,ARHGDIA,ARNTL2,BICD2,C11orf68,C8orf55,CA8,CAV1,CCDC41,CCND1,CCNE2,CENPB,CHST11,CLN8,CNN3,CNOT3,COL4A1,COL4A2,COL5A1,CPM,CSRNP2,CYP1B1,DCAF17,DCBLD2,DEGS1,DNAJB6,DUSP5,DUSP6,DVL1,EFHD2,EHD1,EIF5,ELL2,EML1,EPHB2,EXT1,F2R,F2RL1,FA2H,FAT1,FBXL5,FEM1B,FERMT2,FGF2,FGFR1,FOSL1,FOSL2,FST,GAS2L1,GPR125,GPR172A,GTF3C4,GTSE1,H2AFX,HIP1,HOMER3,HSPA1B,IGFBP1,ITGA3,JUN,KIAA1199,KLF4,KLF6,LHFP,LMO4,LSR,MAFF,MAP3K14,MAP4K5,MAP7D1,MAPKAPK2,MBD4,MDFIC,MFSD9,MICAL2,MICALL1,MREG,MRPS12,MSX1,NFKB2,NFKBIA,NFYA,NKX3-1,NOV,NPAS2,NRP2,P2RY2,PALM2,AKAP2,PANX1,PAQR5,PCGF3, PDSS1,PHLDA1,PHLDA2,PLAUR,PLCL2,PMEPA1,PODXL,POLRMT,PPIF,PPP3CC,PPP3R1,PVRL3,QKI,RAB11FIP1,RAB35,RALA,RAP2A,RBPJ,RELB,RGS20,RHOF,ROD1,SEC14L1,SEC24A,SEMA3C,SENP3,SFRP1,SGK1,SGMS1,SKP2,SLC26A2,SLC31A2,SLC39A7,SLC7A6,SMURF2,SOCS2,SOCS5,SOD2,SOLH,SOX4,SOX9,SPOCK1,SRD5A1,SRRT,SS18,STK10,SYNJ2,TAOK2,TBX3,TGFA,TGFB2,THBD,TLE4,TNFRSF12A,TRIB1,TRIO,TSC22D2,TSPAN5,TTPAL,TUBB2A,TUBB2B,TUBD1,VANGL1,VEGFC,WWTR1,ZBED4,ZBTB1, ZFP36L1,ZNF131,ZNF174,ZNF557 |
