# Supplementary material for: PrtT-Regulated Proteins Secreted by Aspergillus fumigatus Activate MAPK Signaling in Exposed A549 Lung Cells Leading to Necrotic Cell Death
Source: PLoS One. 2011 Mar 11;6(3):e17509. doi: 10.1371/journal.pone.0017509 (PMC3055868; doi:10.1371/journal.pone.0017509)
Supplement: Text S1 — (DOC) [file pone.0017509.s009.doc]

**Supplement: Computational analysis of microarray**

**Data notation**

We analyzed three data sets. The first data set was aimed to measure the difference between the effect of infection by culture filtrates (CF) of the wild type AF and by a mutant without the PrtT gene. We denote this data set as WT-CF1 vs. PrtT-CF. The second data set was aimed to measure the difference between the effect of the wild type AF culture filtrates and the wild type AF germinating conidia (GC). We denote this data set as WT-CF2 vs. WT-GC. Finally the third data set was aimed to assess the differences between the germinating conidia of the PrtT mutant and the conidia of the wild type AF. We denote this data set as WT-GC vs. PrtT-GC. All profiles were measured in the same human lung cell line.

Unless written otherwise all conditions were repeated three times.

WT-CF vs. PrtT-CF profiles:

1. WT-CF1- infection with filtrates of wild type AF.
2. Ctrl1- uninfected cells (control)
3. PrtT-CF- infection with filtrates of PrtT AF mutant.

WT-CF vs. WT-GC profiles:

1. Ctrl2- uninfected cells (control)
2. WT-GC- infection with conidia of WT AF.
3. WT-CF2 – infection with filtrates of WT AF.

WT-GC vs. PrtT-GC profiles:

1. WT-GC2 - infection with conidia of WT AF.
2. Ctrl3- 2 repeats of uninfected cells (control)
3. Beads – 2 repeats of uninfected cells into which inert beads were inserted (another control mimicking the effect of the cell disruption).
4. PrtT – GC - infection with conidia of PrtT AF mutant.

All profiles underwent preprocessing and filtering (see Low level analysis below)

**Data Analysis**

**WT-CF vs. PrtT-CF data set**

For each bipartition of the conditions we defined a gene as a differential gene if it was found to have significantly different distributions in the two sets, using a two tailed T-test with FDR[2] correction of 0.1, and if the fold change between the averages of the profiles exceeded 1.5.

We created the following gene groups:

WT-CF1 and PrtT-CF vs. Ctrl1: The set of differential genes between WT-CF1 and Ctrl1 contained 1667 probes (covering 1318 genes, by probe to Entrez mapping). The set of differential genes between PrtT-CF and Ctrl1 contained 2213 probes (1851 genes). The intersection of the two sets contained 1004 probes, covering 812 genes. We call this set the common signal (CS) set.

The CS set can be divided into two sub-groups of up-regulated and down-regulated genes (compared to Ctrl1). We obtained this grouping by applying the KMeans algorithm with k=2 (average homogeneity 0.928, average separation -0.968, other k's obtained inferior clustering results). In general the partition is consistent between the PrtT-CF profiles and WT-CF1 profiles – most of the genes that are up regulated in the WT-CF1 are up-regulated in the PrtT-CF treatment, and the same holds for down-regulated genes. The down regulation cluster contained 299 probes (262 genes) and the up-regulation cluster contained 705 probes (550 genes).

WT-CF1 vs. PrtT-CF Unique signal groups: When selecting differential genes between the WT-CF1 group and the PrtT-CF group, the result was a set of 776 probes covering 699 genes, and we call this set the unique probes set. We clustered the selected probes into four clusters using the SOM algorithm [6] using a grid layout of 2X2. Each gene's profile in the input matrix was standardized, the average homogeneity was 0.912 and average separation was -0.41. The results are shown in SFigure4. The two largest clusters correspond to up and down regulation in PrtT-CF. Cluster1 holds most of the up-regulated probes in PrtT-CF and cluster 4 holds most of the down-regulated probes in PrtT-CF. Finally we compared this clustering to partitioning the probes set to up-regulated in PrtT -CF (517 probes) and down-regulated in PrtT (259 probes). This partitioning yields average homogeneity of 0.833 and average separation of -0.969, therefore for further analysis we used this simple partitioning and not the SOM solution.

We tested each of the probe groups for GO annotations enrichment the TANGO [4] algorithm, (with FDR correction of 0.1). For enrichment analysis of known Human transcription factors we used the PRIMA [3] algorithm (minimum accepted P-value was 10E-4). Finally, for enrichment of KEGG pathways [5] we used a simple hyper -geometric test (minimum accepted P-value was 10E-4). The enrichment tests and the clustering algorithms (Kmeans, SOM) are implemented in the Expander program for gene expression analysis [1].

**WT-CF vs. WT-GC data se**t

Comparison with Ctrl2 group: We compared the WT-CF2 conditions with Ctrl2 and the WT-GC conditions with Ctrl2. In each comparison we defined a gene as differential if it was found significant in a two tailed T-test with FDR correction 0.2, and if the fold change between the averages of the gene exceeded 1.5 (The FDR threshold was increased to fit the different microarray type). For the WT-CF2 and controls comparison 753 probes covering 615 genes were selected, 396 down-regulated (324 genes) in WT-CF2 vs. Ctrl2 and 357 (291 genes) up-regulated. For the WT-GC vs. Ctrl2 comparison 116 probes (95 genes) were selected, 115 up-regulated (94 genes) in WT-GC vs. Ctrl2 and 1 down-regulated.

WT-CF2 and WT-GC comparison: For the comparison of the WT-CF2 and the WT-GC conditions, only one probe passed the T-test and fold change criteria. Therefore we used a simple threshold rule, selecting probes with at least 2 fold change. A set of 329 probes (266 genes) were found. We clustered the selected probes using all of the experiment's conditions, in order to take into account these probes expression in the control condition as well. We used the K-means algorithm with K=4. Each gene's profile in the input matrix was standardized. The average homogeneity was 0.934 and the average separation was -0.26. The clustering results are shown in SFigure5.

The same enrichment tests for the first data set were applied.

**WT-GC vs. PrtT-GC data set**

Differential genes between Beads and Ctrl3

Using T-test with FDR correction of 0.1, only one probe was detected as differential. Using fold change filter of 2 between the average expressions of probes, only 9 probes remain and only one of them can be mapped to a gene.

Based on this result, in all the experiments below we considered both beads and control as control, denoted as Beads&Ctrl3.

Differential genes between PRTT and Beads&Ctrl3

Using T-test with FDR correction of 0.1, only one probe was detected as differential. Using fold change filter of 2 between the average expressions of probes, only 19 probes remain and only 7 can be mapped to genes.

Based on this result, we conclude that only very minor difference can be found between the PrtT-GC experiment and the controls.

Differential genes between WT-GC2 and PrtT-GC

Using a two tailed T-test with FDR correction of 0.1, only 4 probes (4 genes) were detected, of which only 3 have fold change of at least 1.5.

Based on this result, we conclude that only very minor difference can be found between the PrtT-GC experiment and WT-GC2.

Differential genes between WT-GC2 and Beads&Ctrl3

Using a two tailed T-test with FDR correction of 0.1, and fold change threshold of 2 between the averages of the profiles, 1217 probes (1031 genes) were selected, of which 319 were up-regulated in WT-GC2 (229 genes) and 898 were down regulated (802 genes).

Inconsistency between WT-GC vs. WT-CF2 and WT-GC vs. PrtT-GC experiments

We compared WT-GC conditions vs. control in two different experiments. Surprisingly, in the WT-GC vs. PrtT-GC experiment the number of differential probes was drastically higher than in the WT-GC v. WT-CF2 experiment (1217 and 116 respectively). However GO annotation analysis in the WT-GC v. WT-CF2 found 62 enriched GO annotations (all of them in the up-regulated group) while only 40 were found in WT-GC vs. PrtT-GC (of which 10 are in the up-regulated group). It seems that the WT-GC vs. PrtT-GC experiment suffers from extremely low specificity in terms of GO annotation enrichment. A possible reason is high noise level in the WT-GC vs. PrtT-GC data set, manifested as high variance. Still, we find it hard to explain these differences, in particular in the enrichment of the down-regulated probes.

For further comparison between WT-GC and controls we used only results from WT-GC vs. WT-CF2 experiment.

**Low level analysis**

WT-CF1 vs. PrtT-CF

1. Using Affymetrix Expression Console we normalized the probes using RMA and Quantile, taking into account detection calls.
2. Genes with expression values lower than 5.5(log2 scale) across all conditions were filtered out, leaving 14521 probes.
3. Hierarchical clustering analysis (SFigure1) implies that the PrtT-CF repeat 3 profile is not similar to the other PrtT-CF repeats (see details below), and was therefore removed from further analysis.
4. We repeated stages 1-2 without PrtT-CF repeat 3, leaving 14831 probes.

Hierarchical Clustering was performed twice; one analysis was based on all probes in the data set and the second was based on 1000 probes with highest variation. Results are shown in SFigure 1. In general the resulting trees are consistent. The profiles are divided to two main groups: control repeats group and the treatment repeats group. In the second group, the PrtT-CF third repeat is closer to the WT-CF1 (denoted as 'AF' in SFigure 1) sub-group than to the PrtT-CF (denoted as 'PrtT' in SFigure 1) treatment group. Therefore in further analysis performed in this study we excluded this repeat.

WT-CF2 vs. WT-GC

1. Using Expander we normalized the probes matrix (GC-RMA), taking into account detection calls.
2. Using quantile normalization in Expander we normalized the probes matrix.
3. Genes with expression values lower than 5.5(log2 scale) across eight or all nine conditions were filtered out, leaving 14421 probes in the data set.
4. WT-GC repeat 1 and WT-CF2 repeat 3 were removed, as these repeats showed significantly low hybridization values, this preprocessing was done in two steps:
5. Using the complete matrix from stage 3, we computed in each repeat the number of probes that showed value less than 5.5. In WT-GC repeat 3 there were 3756 such probes, over four times more than in the other repeats (SNTable 2. Compare SNTable 1 for the previous experiment). Such large differences are result of low intensities flooring done by Expander.
6. We repeated stages 1-3 without the WT-GC repeat 3 and computed the same values (results inSNTable3). Hierarchical clustering analysis revealed that WT-CF2 repeat 3 acts as an out-group of the other WT-CF2 samples, as shown in SFigure2.This repeat was therefore removed.
7. We repeated stages 1-3 without the removed conditions. Stage 3 was slightly modified to removing genes with values lower than 5.5 across all conditions only, leaving 14653 probes.

WT-GC2 vs. PrtT-GC

1. Using Affymetrix Expression Console we normalized the probes (using RMA).
2. Using Expander we normalized (Quantile) the matrix.
3. Filtering: genes with intensities lower than 5 (log 2 scale) in all conditions were filtered out, leaving 16508 probes in the data after the filtering.
4. Hierarchical clustering analysis (SFigure 3) shows inconsistency between the repeats of the experiment. For example, repeat 2 of Ctrl3 clusters together with the PrtT-GC conditions. One drastic inconsistency was of repeat 3 of the WT-GC2 condition, which clustered with the PrtT subtree, and far from the other two WT-GC2 repeats. Therefore we decided to remove this condition and repeated steps 1-3 without it. The new matrix contained 14575 probes.

| WT-CF1:1 | WT-CF1:2 | WT-CF1:3 | Control1 | Control2 | Control3 | PrtT-CF1 | PrtT-CF2 | PrtT-CF3 |
| --- | --- | --- | --- | --- | --- | --- | --- | --- |
| 1559 | 1559 | 1559 | 1560 | 1560 | 1559 | 1560 | 1559 | 1559 |

SNTable 1: (WT –CF1 vs. PrtT-CF preprocessing) after filtering in Step 2 of preprocessing. For each repeat the number of probes with values lower than 5.5 is computed.

| WT-GC1 | WT-GC2 | WT-GC3 | Control1 | Control2 | Control3 | WT-CF2:1 | WT-CF2:2 | WT-CF2:3 |
| --- | --- | --- | --- | --- | --- | --- | --- | --- |
| 3756 | 842 | 865 | 845 | 843 | 846 | 856 | 863 | 905 |

SNTable 2: (WT-CF2 vs. WT-GC) Step 4.1 of preprocessing. For each repeat the number of probes with values lower than 5.5 is computed.

SNTable 3: (WT-CF2 vs. WT-GC) Step 4.2 of preprocessing, for each repeat the numbers of probes with values lower than 5.5 is computed.

| WT-GC1 | WT-GC2 | Control1 | Control2 | Control3 | WT-CF2:1 | WT-CF2:2 | WT-CF2:3 |
| --- | --- | --- | --- | --- | --- | --- | --- |
| 658 | 653 | 657 | 660 | 654 | 623 | 646 | 704 |


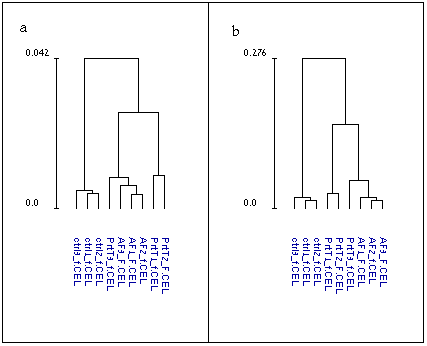


SFigure1: Hierarchical clustering analysis of WT-CF vs. PrtT-CF data set ; a) based on all 14521 probes in the data set; b) based on 1000 probes with highest variation. WT-CF1 is denoted as 'AF', PrtT-CF is denoted as 'PrtT'.


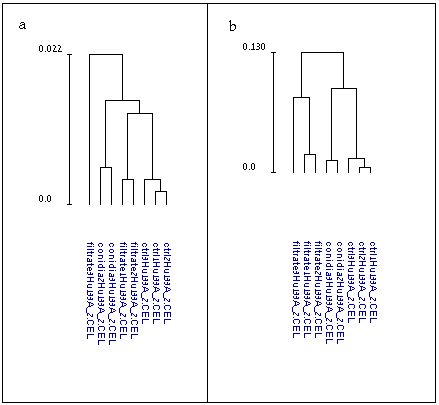


SFigure2: Hierarchical clustering analysis on the data produced in Step 4.2 of preprocessing of WT-CF vs. WT-GC data set; a) based on all 13847 probes in the data set, the third repeat of the WT-CF2 acts as an out-group for all other conditions. WT-GC is denoted as 'conidia' and WT-CF2 is denoted as 'filtrate'; b) based on 1000 probes with highest variation, the third repeat of the WT-CF2 experiment clusters separately from the other filtrate experiments, with very long branch to the lowest common ancestor in the tree.


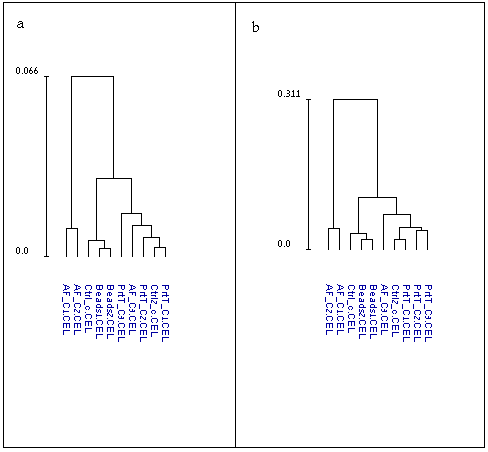


SFigure3: Hierarchical clustering of WT-GC vs. PrtT-GC data set before removing the problematic condition; a) based on all 16508 probes in the data set. WT-GC2 is denoted as 'AF' and PrtT-CF is denoted as 'PrtT'; b) based on 1000 probes with highest variation.


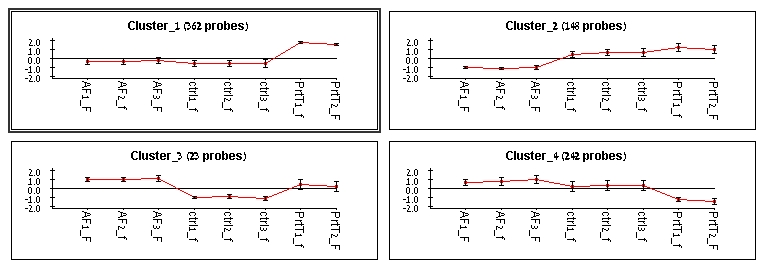

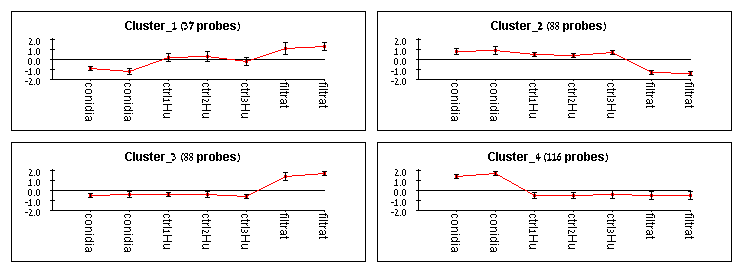
 SFigure5: Clustering of the differential probes between the WT-GC and the WT-CF2 conditions in the WT-CF vs. WT-GC data set, This clustering was obtained using the Kmeans algorithm with K=4. WT-GC is denoted as 'conidia' and WT-CF2 is denoted as 'filtrate'

SFigure4: WT-CF vs. PrtT-CF data set; clustering analysis of the WT-CF1 vs. PrtT-CF unique resonse. The probes were devided to groups with all of the data set's conditions. WT-CF is denoted as 'AF', PrtT-CF is denoted as 'PrtT'.

References

| [1]Expander: from expression microarrays to networks and functions. |
| --- |
| I. Ulitsky, A. Maron-Katz, S. Shavit, D. Sagir, C. Linhart, R. Elkon, A. Tanay, R. Sharan, Y. Shiloh, R. Shamir. *Nature Protocols vol5, pp 303-322, (2010).* |
| [2] Benjamini, Y. & Hochberg, Y. Controlling the false discovery rate: a practical and powerful approach to multiple testing. *Journal of the Royal Statistical Society. Series B (Methodological)* 289–300 (1995). |

[3] Elkon, R., Linhart, C., Sharan, R., Shamir, R. & Shiloh, Y. Genome-wide *in silico* identification of transcriptional regulators controlling the cell cycle in human cells. *Genome Res.* **13**, 773–780 (2003).

[4] Shamir, R. *et al.* EXPANDER—an integrative program suite for microarray data analysis. *BMC Bioinformatics* **6**, 232 (2005).

[5] Kanehisa, M. & Goto, S. KEGG: Kyoto encyclopedia of genes and genomes. *Nucleic Acids Res.* **28**, 27–30 (2000).

[6] T. Kohonen. Self-Organizing Maps. Springer, Berlin (1997).
